# SUPPORT EXCHANGES IN COUPLES COPING WITH EARLY-STAGE ALZHEIMER’S DISEASE

**DOI:** 10.1093/geroni/igad104.0058

**Published:** 2023-12-21

**Authors:** Meng Huo, Kyungmin Kim

**Affiliations:** University of California, Davis, Davis, California, United States; Seoul National University, Seoul, Republic of Korea

## Abstract

People living with Alzheimer’s disease (AD) typically receive considerable support from their spousal caregivers. Yet, we know little about whether people with early-stage AD may still be able to help their caregivers and how potential support exchanges in these couples may vary depending on the quality of their relationships. We drew on dyadic data from 37 older couples in which one partner lives with mild-to-moderate AD (Mage = 77yrs, 48% male) and the other serves as a spousal caregiver (Mage = 76yrs, 53% female). Couples participated in concurrent interviews during which they each reported on a variety of demographic and relationship characteristics, such as relationship quality, and couple support exchanges. People with early-stage AD perceived their relationships with spousal caregivers as more positive and less negative than did these caregivers. Support did not flow unidirectionally in these caregiving couples, especially not among those in better-quality relationships. Specifically, caregivers who viewed their relationships as more positive/less negative considered it less stressful to provide emotional and practical support to their partners with AD. Patients who viewed their relationships as more positive/less negative provide more frequent emotional and practical support to their caregivers and also found such support provision more pleasant and less stressful. Findings reveal positive contribution that older adults with dementia, who have been solely treated as recipients of care, can make and shed light on the development of dyadic interventions by highlighting the importance of mutually beneficial relationships in early stages of AD.

